# General Self-Efficacy and Employability Among Financially Underprivileged Chinese College Students: The Mediating Role of Achievement Motivation and Career Aspirations

**DOI:** 10.3389/fpsyg.2021.719771

**Published:** 2022-01-21

**Authors:** Dan Wang, Danyang Guo, Chao Song, Lianming Hao, Zhihong Qiao

**Affiliations:** ^1^School of Education Science, Jilin Normal University, Siping, China; ^2^Faculty of Psychology, Beijing Normal University, Beijing, China; ^3^Faculty of Education, Beijing Normal University, Beijing, China; ^4^Faculty of Psychology and Educational Sciences, Ghent University, Ghent, Belgium; ^5^School of Mathematics, Jilin Normal University, Siping, China

**Keywords:** college students, self-efficacy, employability, achievement, motivation, career, aspirations

## Abstract

Although previous research shows that general self-efficacy is related to employability, the mechanism of them is unclear. Thus, this study aims to explore the relationship between general self-efficacy and employability, examines the mediating role of achievement motivation and career aspirations among financially underprivileged college students in China. The analysis of 651 participants (59% female, 41% male) from six provinces indicates that general self-efficacy positively predicts employability through the mediating chain of achievement motivation and career aspirations. Based on these findings, the researchers propose feasible suggestions for related issues of financially underprivileged college students and future research.

## Introduction

Scholars from several disciplines have focused on the development of students’ employability skills. Crucial factor of employability directly affects the success of university students in employment (Lau et al., 2014), and is a core competency to secure students a job (Harvey, 2001). Research has confirmed the importance of improving students’ employability (Sin et al., 2017) to facilitate their successful employment (Gbadamosi et al., 2015). As internal factors (Abele and Spurk, 2009; Millard, 2020) affect the development of students’ employability, therefore, this study further explores the internal determinants of university students’ employability.

Studies at financially underprivileged college student groups (Hu et al., 2020) found that employability is a key factor that enables students to achieve career success regarding career choice and access to employment opportunities (Flores et al., 2017; Eimer and Bohndick, 2021). In China, financially underprivileged college students are identified via the document “Guidance on Carefully Identifying Students from Families with Economic Difficulties in Higher Education” (Education and Finance No. 8, 2007). It is jointly issued by the Chinese Ministry of Education and the Ministry of Finance, stating that students from families with economic difficulties are those who struggle to cover their basic study and living expenses during their school years with the resources available to them and their families. Researchers define financially underprivileged college students based on their family’s financial income, providing that most are unable to afford university-related expenses, which means financial difficulties in maintaining the normal study and living expenses at their institution (Zhang et al., 2021). Several studies have suggested that compared to non-financially underprivileged college students, financially underprivileged college students show low self-confidence and poor employability (Cheng and Dong, 2016; Zhang and Chang, 2019; Gan and Wang, 2021). Indeed, employability is crucial for financially underprivileged college students to escape poverty through gainful employment, but only a few studies have examined the antecedent variables of employability and their development mechanisms, therefore, this study will examine the underlying mechanisms of employability enhancement among that group.

According to Bandura (1977) social cognitive theory (SCT), personal attributes, environmental influences, and intentional behaviors are interlinked (Cupani et al., 2010). That is, personal behaviors are formed through the interaction between personal thoughts and environmental emotions (Peng et al., 2018). Based on SCT, social cognitive career theory (SCCT) clarifies the relationship between influences in occupational domain and career development (Lent et al., 1994; Lent and Brown, 2006; Burga et al., 2020), which focuses on individuals’ ability to shape their occupational behaviors. Self-efficacy refers to a student’s beliefs about their successful performance, education-related behaviors and abilities, which is an important factor to initiate spontaneous motivation and engagement in learning (Parker et al., 2006). A lack of self-efficacy poses a barrier to successful integration into a profession, for example, some graduates have difficulty in successfully embarking on their careers (Swanson and Fouad, 2015). As there is a lack of research applying SCCT theory to general self-efficacy and employability, this study attempts to based on that to explore the mechanisms of general self-efficacy on employability.

Moreover, in SCCT, achievement motivation is an internal drive for individuals to pursue excellence and strive for success, which motivates people to act (Yeh et al., 1992). In addition, career ambition is a protective factor for employability, and plays a crucial role in career development behavior so that influences career development (Super et al., 1990). Related studies have shown that higher levels of career ambition is associated with higher employability (Gbadamosi et al., 2015). However, previous studies have neglected general self-efficacy, achievement motivation, career ambition and employability in applying SCCT theory to the mechanisms of employability among college students. Therefore, this study aims to explore how the general self-efficacy of financially underprivileged college students influences achievement motivation, which subsequently affects career aspirations and employability, based on the pathway construct of employability formation provided by SCCT.

## Literature Review

### Theoretical Background of Social Cognitive Career Theory

According to SCCT, the core elements that drive career/employment behaviors are self-efficacy, outcome expectations, and choice of goals (Lent et al., 1994), the chain among these elements further addresses how individuals achieve career development success. Thus, SCCT forms the theoretical basis as assessing self-efficacy for employability in this study (Lent et al., 1994; Liu et al., 2020; Zhao et al., 2021).

SCCT is an empirically validated model that has been widely accepted (Brown et al., 2011; Duffy et al., 2014; Burga et al., 2020). According to Lent et al. (1994), self-efficacy is the key component of SCCT and directly affects behavior (Brown et al., 2011; Duffy et al., 2014). Outcome expectation represents a person’s judgment of the consequences resulting from the execution or non-execution of a specific behavior (Brown et al., 2011; Caesens and Stinglhamber, 2014; Duffy et al., 2014). According to SCCT, self-efficacy helps to determine outcome expectations. They are both precursors to goals and jointly lead to choice goals (Lent et al., 1994).

Although various SCCT studies have included outcome expectations, the operationalization of that structure has varied. Many of the measures used are based on success-related expected outcomes in a specific domain, particularly in the form of self-outcomes (e.g., intrinsic motivation or rewards; see Lent and Brown, 2006). The pattern of manifestation of outcome expectations can be embodied as an achievement motive and the goals represented by career aspirations can directly impact behavior.

Specifically, the theory is based on the core cognitive variable of self-efficacy, which facilitates the establishment of choice goals, and thus, choice behavior through the role of outcome expectations. However, the mechanisms by which general self-efficacy affects employability been overlooked in previous studies Therefore, this study aims to explore the relationship between general self-efficacy and the employability of financially underprivileged college students, through the mediating role of achievement motivation and career aspirations.

### Employability

Employability is a key factor for individuals in the labor market (Fugate et al., 2004), as universities and individuals are interested in improving the employability of graduates, it has received significant attention in higher education. Fugate et al. (2004) refer to employability as a person’s ability to identify and realize career opportunities. Employability is a personal characteristic (Fugate et al., 2004; Vermeulen et al., 2018; Shahzad et al., 2020) that describes the capability of a person to become and remain employable.

Most studies on the employability of financially underprivileged college students have focused on financial support (Castleman and Long, 2016; Melguizo et al., 2016; Zhang et al., 2021) and mental health (Lynch et al., 2000; Dixon and Kurpius, 2008; Cheng and Zhang, 2018). On the one hand, several studies have discussed the impact of mental toughness and positive psychology on individual career development (Wang et al., 2021; Xue, 2021). On the other hand, there were studies indicating that externally acquired assistance, such as student loans, fail to fundamentally extricate these students from poverty (Huang et al., 2017). Considering numerous researches have provided significant effects of intra-individual factors, such as career self-efficacy, on employability (Magagula et al., 2020), therefore, this study aim to explore the impact of intra-individual factors on employability among financially underprivileged college students.

Researchers Pan and Lee (2011) focused on measuring employability from the perspectives of general ability, professional ability, work attitude, career planning ability, and confidence. Numerous researchers developed employability measurement tools for Chinese college students according to their characteristics, of which largely focused on the aspect of meeting job search needs. Specially, Wang (2006) developed an employability scale for college students comprising four dimensions as self-awareness, communication and cooperation, cognitive ability, and individual reliability, which fits the characteristics of the sample in this study. Thus, this study adopts Wang Yuan’s employability scale.

### General Self-Efficacy

The personal perception of efficacy may further determine the types of activities chosen, the effort to be expended, and the degree of persistence in the effort (Bandura, 1977). In terms of theoretical foundations, the self-efficacy theory emphasizes that the stronger the individual’s belief in their ability to perform a set of actions, the more likely they will initiate and persist in the given activity. In terms of relevant empirical research, Ferris et al. (2017) confirmed the positive link between self-efficacy and related outcomes by several meta-analyses, such as work performance, athletic performance, and academic accomplishment. Accordingly, Self-efficacy can be general or task-specific, allowing individuals to have a range of simultaneous self-efficacy beliefs.

General self-efficacy beliefs mirror the definition provided by Bandura (1977), “the belief in one’s capabilities to organize and execute the courses of action required to manage prospective situations.” General self-efficacy, which is unspecific, concerns an individual’s self-belief that they can complete any set task at any time. A previous study found that individuals’ role breadth self-efficacy was positively related to their perceived employability (Hanzla et al., 2019). Besides that, researchers have explored the relationship between self-efficacy and employability using Chinese postgraduate students as subjects and suggested that self-efficacy positively predicted employability (Zhong et al., 2020). Moreover, Yan et al. (2019) found that university students’ self-efficacy in career decision-making positively predicted employability.

Research has found that college students’ self-efficacy during job searches positively predicts employment outcomes (Moynihan, 2003). For example, job seekers with low job search self-efficacy tend to adopt ineffective job search techniques and approaches (Wanberg et al., 1999). In addition to that, studies based on SCCT have found a significant positive correlation between self-efficacy and employability (Liu et al., 2020; Zhao et al., 2021). Therefore, we proposed the following hypothesis:

***H1: General self-efficacy positively predicts employability.***

### Achievement Motive

The achievement motivation theory developed by Atkinson and McClelland (Atkinson and John, 1957) defines the tendency to approach an achievement task in terms of two motive factors: the motive to approach success and to avoid failure. The expectancy value theory expands on that idea, proposing that behavior is strongly influenced by an individual’s expectancy of outcome, and the subjective value of that successful outcome (Weiner, 1992). Moreover, research also suggests that the expectancy of success will increase a person’s willingness to overcome challenges and struggles (Eccles and Wigfield, 2002).

According to the above arguments, achievement motivation is an internal drive for individuals to excel and succeed, an internal motivator for action (Yeh et al., 1992), and influenced by general self-efficacy. According to the theory of the role of self-efficacy, individuals with high sense of self-efficacy will mobilize all their strengths to overcome difficulties, which serves a key role in the formation of motivation (Bandura, 1977).

### General Self-Efficacy-Achievement Motive

Moreover, evidence suggests that different levels of self-efficacy influence motivation, with higher self-efficacy leading to higher levels of motivation, and vice versa (Cui et al., 2017). Additionally, individuals with low self-efficacy have lower personal motivation, and consequently adopt less effective job search skills (Wanberg et al., 1999); higher levels of general self-efficacy also links to higher motivation in goals, allowing greater efforts and persistence through difficulties (Bandura and Wood, 1989).

### Achievement Motive-Employability

Furthermore, research has found that higher levels of self-efficacy are associated with higher levels of achievement (Chemers et al., 2001; Torres and Solberg, 2001). Achievement motivation also influences numerous behaviors, especially employability that is significantly impacted (Barron and Harackiewicz, 2001; Diao, 2015). In addition to them, studies based on SCCT suggest that achievement motivation positively predicts academic performance among university students (Caldwell and Obasi, 2010). Therefore, we put forth the following hypothesis:

***H2: General self-efficacy positively predicts employability through the mediating role of achievement motivation.***

### Career Aspirations

Career aspirations are an individual’s goals and expectations for a particular career that determine an individual’s career choice (Gottfredson, 1981). Additionally, career aspirations also can be defined as “an individual’s expressed career-related goals or choices” (Rojewski, 2005) and the significant predictor of later occupational attainment (Holland and Lutz, 1968). Hence this study operationalizes the concept of career aspirations as the individual’s career goals, which is approved by relevant previous researches that it predict future career choices and achievements (Schoon and Polek, 2011).

Moreover, numerous well-established factors influence career aspirations, including family socioeconomic status (SES), ethnicity, and gender. For example, teenagers from higher-income families reported greater intentions to pursue professional careers and continue their education than teenagers from lower-income families (Ashby and Schoon, 2010; Mau and Bikos, 2011), further, college students and females tend to report lower career ambitions than men (Danziger and Eden, 2007).

### General Self-Efficacy—Career Aspirations

Career ambition is seen as an indicator of students’ future career success (Drai et al., 2018). Mau and Li (2018) found that self-efficacy positively predicted students’ career ambitions. Further, individuals with high levels of self-efficacy tend to have strong levels of ambition, that is, self-efficacy predicts the strength of ambition (Super et al., 1990). Additionally, research on disadvantaged youth’s career aspirations who are in low SES also emphasized the effect between general self-efficacy and career aspirations (Direnzo et al., 2013). Thus, it can be seen that the positive effects of general self-efficacy and various task efficacies on work and career outcomes have been addressed, the latter specifically including job performance and career success (Locke et al., 1984; Saks, 1995; Abele and Spurk, 2009).

### Career Aspirations—Employability

Teenage ambition predicts adult occupational attainment (Mello, 2008) and future occupational status (Schoon and Parsons, 2002). Simultaneously, career ambition is a protective factor for employability. That is, ambition plays a vital role in career development behavior and influences individual career development (Super et al., 1990). Further, higher levels of career ambition are associated with higher employability (Gbadamosi et al., 2015), with career ambition positively predicting employability (Drai et al., 2018).

### General Self-Efficacy—Career Aspirations—Employability

Numerous researches have shown that career self-efficacy is a positive predictor of career aspirations (Hartman and Barber, 2019). Specifically, when individuals are more confident, they persist in overcoming difficulties and tend to adopt behaviors that contribute to their career success (Johnson et al., 2010), which also shows such individuals understand the importance of engaging in behaviors that would help them achieve desired outcomes (Bandura, 1977, 2006). Thus, enhancing individuals’ self-efficacy beliefs affects how they behave and strive toward success (Johnson et al., 2010). Moreover, considering a study using SCCT theory revealed that parental variables influence adolescents’ career ambitions and career behaviors (planning and exploration) through self-efficacy (Sawitri et al., 2014), therefore, the following hypothesis was proposed:

***H3: General self-efficacy mediates the employability relationship through career aspirations.***

### Achievement Motivation—Career Aspirations

In addition, career aspirations may also mediate the relationship between achievement motivation and employability. Career aspirations are closely linked to an individual’s aspirations, beliefs, and achievement motivation, all of which govern behaviors toward goal attainment (Rojewski, 2005). In addition to that, career aspirations are positively related to achievement motivation. And the choice of career goals (career aspirations) are not only significantly influenced by outcome expectations (achievement motivation) (Li, 2005), but also mediate the relationship between achievement motivation and other career adjustment variables (Wigfield et al., 2002).

Researchers define aspirations or career motivation as the extent to which individuals desire promotion and recognition (Peters et al., 2013). Considering expectancies predict occupational ambitions (Nauta and Epperson, 2003), therefore, we put forth the following hypothesis:

***H4: Career aspirations are an essential mediator of achievement motivation affecting employability, and achievement motivation positively predicts career aspirations.***

In summary, this study aims to examine the role of general self-efficacy as a predictor of employability based on SCCT and, analyze the mediating role of achievement motivation and career aspirations in the relation between general self-efficacy and employability.

## Methodology

### Participants and Sampling

Convenience sampling method was employed in this study, recruiting freshmen and sophomore students from nine colleges and universities, including Beijing United University, Inner Mongolia Agricultural University, Jilin Normal University, Inner Mongolia University of Finance and Economics, Shenyang University of Technology, Changchun Normal University, Jilin Agricultural University, Anhui University of Science and Technology, and Huaiyin Normal University, from the Jilin, Beijing, Inner Mongolia, Liaoning, Shandong and Anhui Provinces, respectively. Questionnaires were distributed via the Questionnaire Star app, and 2,695 questionnaires were collected. A total of 2,485 questionnaires were valid, yielding a return rate of 92.2%. Based on the definition of financially underprivileged college students in this study, 651 poor university students (26.2% of the total number of students; 59% female; 41%male) were screened according to whether their families applied for financial hardship certificates from their local governments or student loans from their schools.

### Measures

#### Employability

The employability scale, developed by Wang (2006), consists of 23 items that measure four dimensions of employability: cognitive ability, individual reliability, communication and cooperation, and self-awareness. Cognitive ability includes items such as, “I can acquire new knowledge and skills quickly” and “I can reason logically.” These items assess the individual’s understanding and awareness of knowledge, society, and problem areas. The scale is scored on a seven-point Likert scale, with 1 being “completely disagree” and 7 being “completely agree.” Higher scores indicate greater employability. The employability scale displayed good reliability and validity in various studies assessing the employability of college students (Chen and Li, 2011). In this study, Cronbach’s alpha was 0.962.

#### General Self-Efficacy

The general self-efficacy scale was developed by Schwarzer et al. (1997) and revised by Wang et al. (2001). It is a unidimensional scale with 10 items; for example, “I can solve my problems if I do my best” and “I am confident that I can deal effectively with difficulties.” Answers are scored on a four-point Likert scale, with 1 being “completely incorrect” and 4 being “completely correct.” The total score is obtained by summing the scores of all 10 questions and dividing the value by the number of questions, with higher scores indicating higher general self-efficacy. Liang and Su (2011) applied this scale to measure the general self-efficacy of university students. Cronbach’s alpha in this study was 0.892.

#### Achievement Motivation

The Achievement Motivation Scale was developed by Gjesme and Nygard in 1970 and revised by Yeh et al. (1992) It comprises 30 questions divided into two equal subscales of motivation to pursue success and to avoid failure. The success-seeking motivation score minus the failure-avoidance motivation score represents the total achievement motivation score. Items regarding motivation for success include, “I like to persevere in problems that I am unsure I can solve” and “I enthusiastically face issues that I am not sure I can overcome.” Answers were rated on a four-point Likert scale, with 1 being “completely incorrect” and 4 being “completely correct.” Higher scores indicated stronger achievement motivation. The Achievement Motivation Scale exhibited good reliability and validity in various studies assessing college students’ achievement motivation (Shen et al., 2013). Cronbach’s alpha in this study was 0.883.

#### Career Aspirations

The Career Aspirations Scale, developed by Wang (2008), comprises 25 items with six dimensions: job satisfaction, interpersonal relationships, degree of challenge, contribution to society, work environment, and development prospects. Items regarding degree of challenge and job satisfaction include, “My job fosters creativity,” “My job has high social status,” and “My job is challenging.” Answers are rated on a five-point Likert scale, with 5 indicating “extremely important” and 1 indicating “unimportant.” Higher scores indicated greater importance attached to the occupational characteristics represented by the item. Chen and Li (2011) applied this scale to examine career ambition. In this study, Cronbach’s alpha was 0.913.

#### Data Processing

IBM SPSS Statistics for Windows, Version 20.0 and Mplus 7.0 were used for assessing internal consistency, descriptive statistics, and correlation analyses of the scales. SPSS Process components were used for chain mediation tests and bootstrap analysis.

## Results

### Common Method Deviation Test

The Harman one-factor approach was used to test for common method bias, and exploratory factor analysis was conducted regarding the four study variables. The results revealed 15 factors that could be analyzed with a characteristic root greater than 1. The first common factor had an explanatory rate of 23% (< 40%), which indicated that there was no serious problem of common method bias in this study.

### Differences in Demographic Variables Among Financially Underprivileged College Students

Demographic characteristics including gender, major, and year of study were used as grouping variables. Independent sample *t*-tests and ANOVA were conducted on employability, career aspirations, general self-efficacy, and achievement motivation among financially underprivileged college students (Table 1).

**TABLE 1:** Financially underprivileged college students’ basic information table for each variable (*n* = 651).

Characteristic variables	Category	Employability	Career aspirations	General self-efficacy	Achievement motivation
		
		*M* ± *SD*	*M* ± *SD*	*M* ± *SD*	*M* ± *SD*
Gender	Male	5.68 ± 0.77	3.75 ± 0.60	2.49 ± 0.59	10.19 ± 0.69
	Female	5.46 ± 0.71	3.74 ± 0.55	2.40 ± 0.51	10.13 ± 0.62
	*t*	3.807***	0.396	1.866	1.208
Specialties	Arts and history	5.51 ± 0.77	3.75 ± 0.60	2.40 ± 0.56	10.09 ± 0.65
	Science and engineering	5.59 ± 0.72	3.74 ± 0.55	2.46 ± 0.54	10.21 ± 0.64
	*t*	−1.328	0.372	−1.200	−2.259*
Grade	Freshman	5.59 ± 0.73	3.77 ± 0.57	2.45 ± 0.54	10.27 ± 0.69
	Sophomore	5.58 ± 0.72	3.78 ± 0.56	2.39 ± 0.52	10.04 ± 0.56
	Junior	5.44 ± 0.80	3.65 ± 0.60	2.47 ± 0.57	10.06 ± 0.63
	Senior	5.55 ± 0.69	3.71 ± 0.60	2.45 ± 0.63	10.04 ± 0.65
	*F*	1.366	1.792	0.771	6.971***
Class officers	Yes	5.71 ± 0.71	3.82 ± 0.56	2.49 ± 0.58	10.22 ± 0.68
	No	5.42 ± 0.74	3.68 ± 0.57	2.39 ± 0.52	10.10 ± 0.61
	*t*	4.982***	3.252**	2.180*	2.356*
Family residence	Rural	5.53 ± 0.72	3.76 ± 0.54	2.41 ± 0.54	10.14 ± 0.61
	Town	5.57 ± 0.80	3.68 ± 0.63	2.50 ± 0.53	10.22 ± 0.77
	City	5.59 ± 0.77	3.74 ± 0.62	2.46 ± 0.58	10.14 ± 0.63
	*F*	0.352	0.846	1.397	0.787

*p < 0.05, **p < 0.01, ***p < 0.001.

The independent-samples *t*-test showed that employability scores were higher for those who were class officers (*M* = 5.71, *SD* = 0.71) than those not (*M* = 5.42, *SD* = 0.74), *t* = 4.982, *p* < 0.01, and males (*M* = 5.68, *SD* = 0.77) had higher employability scores than females (*M* = 5.46, *SD* = 0.71), *t* = 3.807, *p* < 0.05. Apart from that, other variables were not significantly different (*p* > 0.05).

### Descriptive Statistics and Correlation Analysis

General self-efficacy, achievement motivation, career aspirations, and employability were correlated. Pearson product difference correlation coefficients were calculated to examine the relationship between self-efficacy and employability, and the results showed a significant positive correlation. The specific effects of the correlation analysis are shown in Table 2.

**TABLE 2:** Correlation analysis of the variables of financially underprivileged college students (*n* = 651).

Variables	*M*	*SD*	1	2	3	4
1.Employability(dependent variable)	5.55	0.74	1			
2.Career aspirations(intermediate variable)	3.74	0.57	0.535***	1		
3.General self-efficacy(independent variable)	2.43	0.55	0.406***	0.218***	1	
4.Achievement motivation(intermediate variable)	10.15	0.65	0.413***	0.285***	0.352***	1

*p < 0.05, **p < 0.01, ***p < 0.001.

### The Relationship Between General Self-Efficacy and Employability: A Test of Chain Mediating Effects

General self-efficacy as the independent variable, achievement motivation and career aspirations as mediating variables, and employability as the dependent variable were used to develop a model, hence further explore the relationship among general self-efficacy, achievement motivation, career aspirations, and employability. Since gender and status as a class officer impacted employability, these factors were included as control variables in the structural equation model. The mediating effect was estimated by 5,000 samples with 95% confidence interval according to the sequential test and bootstrap method as recommended by Wen and Ye (2014).

Sequential tests results (Table 3) indicated that general self-efficacy significantly and positively predicted achievement motivation. Achievement motivation and general self-efficacy simultaneously predicted career aspirations, significantly as well as positively. When general self-efficacy, achievement motivation, and career aspirations were included in the regression equation simultaneously, all three had a significant positive predictive effect on employability. Thus, **Hypothesis 1 was validated.**

**TABLE 3:** Regression analysis of the relationship between variables in the chain mediation model.

Dependent variable	Independent variable	Partial regression coefficient				*R*	*R* ^2^	*P*
		β	*SE*	*T*	*P*			
Achievement motivation	Constants	9.14	0.14	64.87	0.00	0.36	0.13	<0.001
	General self-efficacy	0.41	0.04	9.39	0.00			
	Gender	–0.01	0.05	–0.27	0.78			
	Cadres	0.08	0.05	1.62	0.11			
Career aspirations	Constants	1.23	0.35	3.54	0.00	0.33	0.11	<0.001
	Achievement Motivation	0.21	0.04	5.82	0.00			
	General self-efficacy	0.14	0.04	3.25	0.00			
	Gender	0.03	0.04	0.61	0.54			
	Cadres	0.11	0.04	2.59	0.01			
Employability	Constants	0.62	0.36	1.71	0.09	0.66	0.43	<0.001
	Achievement motivation	0.23	0.04	6.13	0.00			
	Career aspirations	0.54	0.04	13.22	0.00			
	General self-efficacy	0.31	0.04	7.17	0.00			
	Gender	–0.15	0.05	–3.28	0.00			
	Cadres	0.12	0.05	2.71	0.01			

*p < 0.05, **p < 0.01, ***p < 0.001.

The direct test for mediating effects (Table 4) revealed that the bootstrap 95% confidence interval for the total indirect effect generated by achievement motivation and career aspirations did not contain a value of 0. That is, both mediating variables had a significant mediating effect on the relation between general self-efficacy and employability.

**TABLE 4:** Chain mediating effects of achievement motivation and career aspirations on the relationship between general self-efficacy and employability.

	Paths	Indirect effect value	Bootstrap SE	Boot LLCI	Boot ULCI	Effect
Total indirect effect		0.213	0.038	0.143	0.289	40.50%
Indirect effect path 1	General self-efficacy—achievement motivation—Employability	0.095	0.019	0.063	0.141	17.95%
Indirect effect path 2	General self-efficacy—Achievement motivation—Career aspirations—Employability	0.045	0.012	0.026	0.075	8.62%
Indirect effect path 3	General self-efficacy—Career aspirations—Employability	0.073	0.031	0.017	0.140	13.92%

*p < 0.05, **p < 0.01, ***p < 0.001.

That mediating role comprises three indirect effects. First, the confidence interval for the indirect effect generated by the general self-efficacy–achievement motivation–employability pathway does not contain a value of 0 (0.095, 17.95% of the total effect), indicating that the indirect effect generated by this pathway was significant. Thus, **Hypothesis 2 was supported.**

Second, the confidence interval for the indirect effect arising from the general self-efficacy–achievement motivation–career aspiration–employability pathway did not contain a value of 0 (0.045, 8.62% of the total effect) and reached a significant level. Therefore, **Hypothesis 4 was validated.**

**FIGURE 1:**
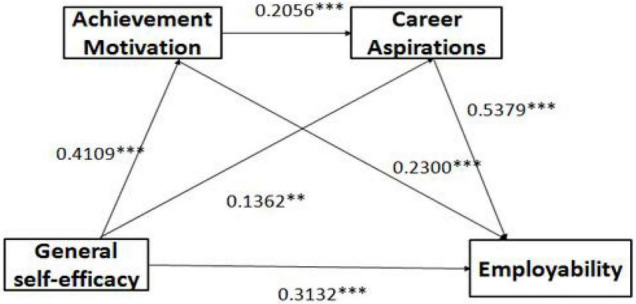
Relationship between general self-efficacy and employability: the chain mediating role of achievement motivation and career aspirations. **p < 0.01, ***p < 0.001.

Finally, the indirect effect of the general self-efficacy–career aspiration–employability pathway had a confidence interval not containing a zero value, indicating that career aspiration had a significant mediating effect between general self-efficacy and employability (0.073, accounting for 13.92% of the total effect). Therefore, **Hypothesis 3 holds true.** The relationship between the variables is shown in Figure 1.

From the above analysis, the relationship among the variables is shown in Figure 1.

## Conclusion

### Discussion

In previous discussions of student employability, various studies have focused on the antecedents of students’ individual behavior patterns and cognition (Cacciolatti et al., 2017; Blázquez et al., 2018). This study takes financially underprivileged Chinese college students as a research sample to verify whether general self-efficacy positively influenced employability, assuming general self-efficacy has a direct effect on achievement motivation, career ambition, and employability in the SCCT model.

Based on our research findings, this study provides the following contributions. First, we validated the applicability of SCCT in China. Specifically, the operational variables of SCCT—outcome expectancy and choice of goal—as well as the role of general self-efficacy on employability, were explored regarding the characteristics of financially underprivileged college students in China. We thus developed a model of the influence mechanism of employability among this population: general self-efficacy influences employability through the chain mediating role of achievement motivation and career ambition. The proposed mechanism of influence of quantitative research results on employability validates the applicability of the SCCT model to a group of financially underprivileged college students in China.

Second, previous researches on SCCT have mostly focused on the influence of environmental factors, with few studies examining the intrinsic psychological mechanisms of individuals (Rasdi and Ahrari, 2020). This study aims to enrich SCCT theory by exploring how the core cognitive variables of self-efficacy, achievement motivation, and career aspirations interact to influence employability among financially underprivileged college students.

Third, most previous studies on SCCT have been conducted from a cross-national or cross-cultural perspective (Alessandro et al., 2018; Zhao et al., 2021), but only a few studies focusing on socioeconomic status (Flores et al., 2017). This study aims to advance SCCT theory and thus provide a direction for future research that focuses on enhancing the employability of financially underprivileged college student groups by SCCT parameters.

The results indicate that there was a significant positive relationship among general self-efficacy, achievement motivation, career aspirations, and employability. Specifically, consistent with existing research, this study confirms that general self-efficacy positively predicted employability (Zhong et al., 2020), via achievement motivation (Chemers et al., 2001; Torres and Solberg, 2001), and also facilitated the establishment of career aspirations (Mau and Li, 2018).

Moreover, the results indicate general self-efficacy can promote employability by enhancing the achievement motivation of underprivileged university students. The higher the general self-efficacy, the more challenging the chosen tasks and the more motivated the individuals (Chi and Xin, 2006). People with high achievement motivation harness their capabilities to improve their self-employability (Cui et al., 2017).

Our results also find that general self-efficacy can influence employability through its role in career aspirations. Individuals’ occupational goals effectively predict their employability and mediate the relationship between general self-efficacy and employability (McArdle et al., 2007). Individuals with strong career aspirations are willing to put in more effort and persist in their actions, all of which are conducive to employability (Gbadamosi et al., 2015).

Besides, this study found that achievement motivation and career aspirations mediated the link between general self-efficacy and employability. That is, the higher the general self-efficacy, the more challenging the chosen tasks and the stronger the motivation (Chi and Xin, 2006). Additionally, achievement motivation was an important factor influencing financially underprivileged college students’ academic and career development (Yang et al., 2016). Moreover, career aspirations served as a predictor for employability (Hasan, 2006).

### Implications

The study offers the following insights as a reference for universities to enhance the employability of underprivileged university students.

First, the results validated Lent et al. (1994) SCCT model which emphasizes the three core concepts of self-efficacy, outcome expectations, and choice of goals as the driving mechanisms that prompt career behavior. We thus demonstrated the importance of the interaction mechanisms of those three core variables among financially disadvantaged university students in China. Further, we confirmed that general self-efficacy influences employability through the mediating role of achievement motivation and career aspirations thus supporting the validation and application of the SCCT in the Chinese context.

Second, our findings provide a pathway for employment guidance among poor university students. This study constructs a model of the mechanisms influencing the employability of financially underprivileged college students from the perspective of their cognitions and, explores the pathway to the formation of their employability. According to our results, universities can enhance the general self-efficacy of financially underprivileged college students to stimulate achievement motivation. Thus, they can establish clear and explicit action goals for job searches to enhance the students’ career ambitions, thereby improving employability. The proposed influence mechanism of employability provides a reference path for colleges and universities to effectively conduct career guidance work.

The third is the enrichment of future research directions. Employability is an important contributor to the career development of underprivileged university students. However, there is a lack of research on their general self-efficacy and employability. Moreover, the indicators of their employability have not received sufficient attention in previous studies, and the mechanism of the role of employability also has not been explored under a more complete system. Our findings can therefore help operationalize the research concept and provide a valid measure of a particular psychological characteristic of the target population through a survey. Furthermore, through the questionnaire method, the data collected can accurately indicate the relationship among the variables and construct a theoretical model of the influence mechanism between the independent and dependent variables.

### Research Limitations

Despite its contributions, this study has several limitations.

First, this study focuses on the influence of intra-individual psychological influences on employability. In future research, the formation of employability among financially underprivileged college students must be explored from the perspective of external influences to comprehensively construct the formation mechanism of employability.

Second, due to time and space constraints, only six universities were considered in this study, with 615 valid questionnaires and an undifferentiated study area. Scholars believe that gender is also an important factor affecting employability; thus, future studies can expand the sample size to improve research representativeness.

Third, this study takes a cross-sectional approach, which recommended subsequent researches to explore the mechanisms of employability through longitudinal studies.

## Data Availability Statement

The original contributions presented in the study are included in the article/supplementary material, further inquiries can be directed to the corresponding author/s.

## Ethics Statement

Ethical review and approval was not required for the study on human participants in accordance with the local legislation and institutional requirements. The patients/participants provided their written informed consent to participate in this study.

## Author Contributions

ZQ played a guiding role in researching. DW mainly took charge of writing. CS and LH mainly took charge of data analysis. DG mainly took charge of language polishing. All authors contributed to the article and approved the submitted version.

## Conflict of Interest

The authors declare that the research was conducted in the absence of any commercial or financial relationships that could be construed as a potential conflict of interest.

## Publisher’s Note

All claims expressed in this article are solely those of the authors and do not necessarily represent those of their affiliated organizations, or those of the publisher, the editors and the reviewers. Any product that may be evaluated in this article, or claim that may be made by its manufacturer, is not guaranteed or endorsed by the publisher.
